# Microbes and associated soluble and volatile chemicals on periodically wet household surfaces

**DOI:** 10.1186/s40168-017-0347-6

**Published:** 2017-09-26

**Authors:** Rachel I. Adams, Despoina S. Lymperopoulou, Pawel K. Misztal, Rita De Cassia Pessotti, Scott W. Behie, Yilin Tian, Allen H. Goldstein, Steven E. Lindow, William W. Nazaroff, John W. Taylor, Matt F. Traxler, Thomas D. Bruns

**Affiliations:** 1 Plant and Microbial Biology, University of California, Berkeley, CA USA; 2 Environmental Science, Policy, and Management, University of California, Berkeley, CA USA; 3 Civil and Environmental Engineering, University of California, Berkeley, CA USA

**Keywords:** Bathroom tiles, Built environment, Chemical ecology, Emissions, Indoors, Metabolites, Microbiota, Stainless steel coupons, Volatile organic compounds

## Abstract

**Background:**

Microorganisms influence the chemical milieu of their environment, and chemical metabolites can affect ecological processes. In built environments, where people spend the majority of their time, very little is known about how surface-borne microorganisms influence the chemistry of the indoor spaces. Here, we applied multidisciplinary approaches to investigate aspects of chemical microbiology in a house.

**Methods:**

We characterized the microbial and chemical composition of two common and frequently wet surfaces in a residential setting: kitchen sink and bathroom shower. Microbial communities were studied using culture-dependent and independent techniques, including targeting RNA for amplicon sequencing. Volatile and soluble chemicals from paired samples were analyzed using state-of-the-art techniques to explore the links between the observed microbiota and chemical exudates.

**Results:**

Microbial analysis revealed a rich biological presence on the surfaces exposed in kitchen sinks and bathroom shower stalls. Microbial composition, matched for DNA and RNA targets, varied by surface type and sampling period. Bacteria were found to have an average of 25× more gene copies than fungi. Biomass estimates based on qPCR were well correlated with measured total volatile organic compound (VOC) emissions. Abundant VOCs included products associated with fatty acid production. Molecular networking revealed a diversity of surface-borne compounds that likely originate from microbes and from household products.

**Conclusions:**

Microbes played a role in structuring the chemical profiles on and emitted from kitchen sinks and shower stalls. Microbial VOCs (mVOCs) were predominately associated with the processing of fatty acids. The mVOC composition may be more stable than that of microbial communities, which can show temporal and spatial variation in their responses to changing environmental conditions. The mVOC output from microbial metabolism on kitchen sinks and bathroom showers should be apparent through careful measurement, even against a broader background of VOCs in homes, some of which may originate from microbes in other locations within the home. A deeper understanding of the chemical interactions between microbes on household surfaces will require experimentation under relevant environmental conditions, with a finer temporal resolution, to build on the observational study results presented here.

**Electronic supplementary material:**

The online version of this article (10.1186/s40168-017-0347-6) contains supplementary material, which is available to authorized users.

## Background

Microorganisms contribute chemicals to their surrounding environment, and these metabolites can have important impacts on ecosystem dynamics. In soil environments, for example, secreted chemicals act as important signaling molecules between microorganisms and have been shown to impact traits such as antibiotic production and virulence [1, 2]. The decomposition of plant material by microorganisms and microbial interactions with living plants imprints on the chemical profiles of belowground, near-ground, and atmospheric chemistry [3–6]. Variation in growth substrate can change the resulting chemicals secreted by microbes, as was observed during the microbial decomposition of leaf litter from different species of plants [7]. Considered from another angle, the identification of microbes themselves by their metabolites has broad application, including in food safety [8], in water quality [9], and in outdoor environments to identify broad ecological functional groups [10]. Just as recent technological advances in sequencing have expanded understanding of the taxonomic composition of microorganisms in different environments, advances in chemical analysis have enabled broader characterization of metabolic products in biological systems, and integrating advances in these fields could help provide insight into the microbiological mechanisms influencing environmental processes and outcomes [11].

The chemical metabolites associated with the human envelope are an active area of research. In the human gut, microbes can modulate the host response to what is ingested [12]. A positive example is how bacteria in the gut synthesize vitamins that are then absorbed into the intestines [13]. However, there was also a case when a major metabolite from bacterial fermentation of an antiviral drug proved lethal to the human hosts [14]. Recently, a 3D cartography of the human skin linked microbiota with its chemical composition [15]. This study showed that the chemicals on the human skin are likely a combination of products from human cells, hygiene products, and microbial metabolism. For instance, the presence of some lipids were highly correlated with the presence of *Propionibacterium*, and the authors showed that *P. acnes* produced one such fatty acid, oleic acid, when grown in culture with triolein [15], a prominent skin lipid [16].

While it is increasingly recognized that human activity is a dominant process structuring the microbial composition in buildings [17, 18], the interplay between microorganisms and chemistry in human-dominated habitats is largely unexplored. An exception to this general feature is seen in water-damaged buildings, where efforts to use chemical tracers to find hidden microbial growth has been assessed, with marginal success to date [19]. Generally, research efforts have focused separately on two central components: biology and chemistry. On one aspect, the application of culture-independent techniques to investigate the microbiology of built environments has led to an expanded view regarding the microbes we encountered indoors and their potential to affect health [20]. In parallel, efforts have been undertaken to better understand the links between exposures to volatile organic compounds in buildings and health [21].

Rarely are the microbiological and chemical signatures of buildings studied together, but there are important reasons to do so. Human residences are distinctive microbial habitats with particular growth substrates (drywall, fibrous insulation, ceramic tiles, etc.), nutrient sources (skin flakes, dust, food and cooking residues), and environmental stressors (soaps and detergents, desiccation, variable temperatures), resulting in complex abiotic and biotic conditions, especially on surfaces. Understanding the microbially mediated chemistry of indoor environments could yield insight into built environments for at least two reasons. First, chemical signatures can be used as general indicators of biologically active microorganisms. Second, microbes interacting with each other, modulated by environmental inputs, could affect the chemical profile of indoor environments. A sound understanding of the microbially mediated influence on indoor air chemistry is lacking but needed [22, 23].

The microbial origin of many chemical agents of interest in the indoor environment are likely surface-bound [24, 25], partly because the area of surfaces and materials in rooms is large, much greater than the superficial area of the room itself. From a microbial perspective, biological and biochemical activity is likely to be concentrated in places that are wet. As such, microbial communities on surfaces that are periodically wetted—such as shower walls, showerheads, sinks, and drains—are distinct from those encountered elsewhere in the home, containing members that include *Methylobacterium* and *Exophiala* [26–30].

As part of a longer-term effort to increase understanding of the microbial ecology of indoor environments, we characterized the microbial composition and associated chemical signatures of periodically wetted surfaces in a home. Applying state-of-the-art chemical methods, we aimed for a preliminary view of both the volatile and soluble compounds associated with these surfaces and to link those molecules with microbes capable of their production. We anticipate that these observations will inform the design of future experimental investigations into the metabolites produced by key members of these communities and how metabolic output is shaped by interspecies interactions and abiotic conditions typical of indoor environments.

## Methods

### Sample collection

Removable surfaces were installed in the kitchen sink and shower stall of an ordinarily occupied residence, left in place for a period of 4 weeks, then removed and analyzed for their microbiological, volatile chemical, or soluble chemical signature. The sampling was conducted at two time points during different seasons.

**Fig. 1:**
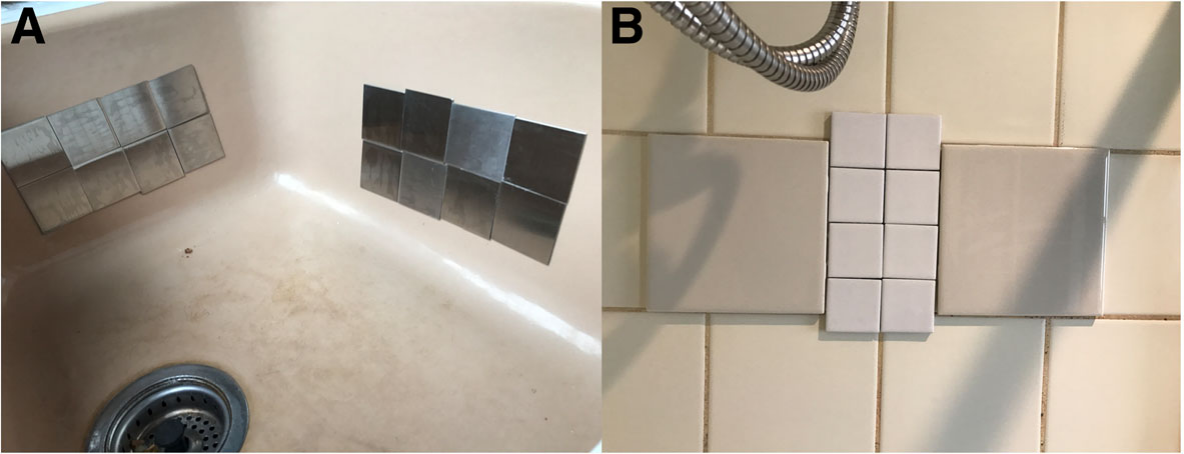
Removable kitchen coupons (**a**) and bathroom tiles (**b**) were installed in an occupied home for approximately 4 weeks, after which time they were analyzed for their microbial and chemical content. There were two clusters of kitchen coupons, referred to as “front” and “side” kitchen sink samples

The samples were collected as part of a larger study exploring the indoor chemistry of residences. Given that the chemical analysis required surfaces to be analyzed in specialized equipment, removable surfaces (shown to correlate with the surface onto which they are attached [31]) were installed in the home (Fig. 1). Occupants of the household were asked to treat the samplers as they would the surface onto which they were attached, with the intention of subjecting the samplers to the typical household environmental exposures.

The study site is a single-family, wood-framed house in Oakland, California. The approximate house age is 80 years. Two adults occupied the home. General household activity and indoor temperature levels were higher during the first sampling campaign than the second (Additional file 1). Samplers were deployed in two locations within the house. In the kitchen, 16 stainless steel coupons (each 25 cm^2^, cut from Alloy 304 sheets; OnlineMetals.com; Seattle, WA, USA) were attached in groups of eight to two separate areas (front and side) in the kitchen sink, which in this particular house was ceramic (Fig. 1a). Similarly, eight 23-cm^2^ ceramic bathroom tiles and two 225-cm^2^ ceramic tiles (Daltile, Dallas, TX, USA) were attached to a shower stall at a height of approximately 1.4 m on the same wall as the shower nozzle (Fig. 1b). Throughout the manuscript, we refer to these samplers as “kitchen coupons” and “bathroom tiles,” respectively.

Some of the analytical techniques were destructive, necessitating different coupons and tiles for the different analysis approaches. As such, the different coupons and tiles were divided among the analytical targets, such that for each sampling period, analyses were based on at least two samples per sampling location. Immediately after installation, surfaces were cleaned with an ethanol wipe.

The first period of sampling, termed sampling 1 or S1, was in August 2016, and the second period of sampling, termed sampling 2 or S2, occurred in February 2017. Upon removal from the residence, samples were processed the same day for each of the respective analyses, as detailed below. Analyses for microorganisms and soluble chemicals were destructive. Analysis for the volatile chemical emissions was not destructive, and these samples were subsequently refrigerated and used for imaging.

### Microbiota analysis

Four kitchen coupons and three bathroom tiles, not including controls, were processed for amplicon sequencing at S1; six kitchen coupons and four bathroom tiles were processed at S2. DNA and RNA were isolated from the sampling surfaces using the Qiagen All Prep DNA/RNA Mini Kit (Redwood City, CA, USA). The surfaces were swabbed with Floq swabs (Copan Diagnostics Inc., Murrieta, CA, USA) moistened in diethylpyrocarbonate (DEPC)-treated water. Cells were disrupted by means of bead beading for 1 min in a tube containing the swab tip, Lysing Matrix D (MP Bio, Burlingame, CA, USA), and 500 μL of Buffer RLT Plus with β-mercaptoethanol. The remaining steps followed the Qiagen kit protocol, except genomic DNA was eluted in 75 μL Buffer EB and RNA was eluted in 35 μL RNAse-free water, both cycled twice through the spin column. The same or following day, RNA was reverse-transcribed to complementary DNA (cDNA) using 5 μL of the extract with the iScript cDNA Synthesis Kit (BioRad, Hercules, CA, USA). DNA and cDNA were then processed in parallel. During sampling 1, water (500 ml) was collected from the kitchen faucet and from the bathroom shower. The water was filtered through cellulose nitrate membrane filter cups (Thermo Fisher Scientific, Waltham, MA, USA), and the filter membrane extracted following the MoBio Power Soil Extraction kit (Qiagen). Controls included sample material (including swabs) and reagent controls as well as positive controls of mock communities, representing a defined mixture of genomic material from different taxa to simulate a microbial community.

For bacterial amplification, we targeted the V4-V5 variable region of the 16S rRNA gene with primers 515F and 806R [32] and barcodes on the reverse primer. DNA was amplified using the HotStarTaq Plus Master Mix Kit (Qiagen, USA) under the following conditions: 95 °C for 5 min, followed by 35 cycles of 94 °C for 30 s, 50 °C for 30 s, and 72 °C for 1 min; after which, a final elongation step at 72 °C for 10 min was performed. PCR amplification reactions contained 0.65 units HotStarTaq, 10× buffer, 200 μM dNTPs, 0.4 μM of each primer, 0.25 μg bovine serum albumin (BSA), 5 or 2.5 μL of sample DNA and cDNA, respectively, and water to a singular 25 μL final reaction volume. PCR products were cleaned using Agencourt AMPure magnetic beads (Beckman Coulter, Brea, CA, USA), quantified with the Invitrogen Qubit™ HS-DS-DNA kit (Invitrogen, Carlsbad, CA, USA), and pooled in equimolar concentrations (10 nM). Fungal communities were characterized by targeting ITS (internal transcribed spacer) 1 spacer of the rRNA gene as previously described [33], except that 5 μL of genomic starting material was amplified in a singular reaction. The MiSeq (2 × 250 PE) libraries were sequenced at the Vincent J. Coates Genomics Sequencing Laboratory in the California Institute for Quantitative Biosciences (QB3) at the University of California, Berkeley.

Bacterial reads were processed using mothur v.1.38.0 [34], and the commands are detailed in Additional file 2. Forward and reverse reads were paired, and in subsequent screening, no ambiguous base calls were allowed; reads with homopolymers exceeding 8 bp and shorter than 270 bp were removed. The data set was dereplicated, and unique sequences were aligned against the SILVA reference database (release 128) containing 168,000 bacterial SSU rRNA sequences [35]. The data set was further denoised by running the “pre.cluster” command [36], and chimeras were removed with the UCHIME algorithm [37], both implemented in mothur. Unspecific amplification products (mitochondria, chloroplasts, Archaea, Eukarya, unknown domain) were removed. The remaining sequences were clustered into operational taxonomic units (OTUs) at sequence divergences of 3% [38] and were assigned taxonomy using the curated SILVA database. Variation in 16S gene copy number across taxa can affect inferences about community diversity [39], but this potential source of bias was not accounted for here. The OTU table was subsampled, and OTUs present in the negative (template-free and extraction) controls were removed from the full OTU table when they were represented by a greater read count in the negatives than in the samples. When the resulting OTU was rarefied to 13,000 sequences per sample, all negative control samples were excluded. OTUs with < 10 total sequences were excluded, based on the mock communities sequence read distribution.

The ITS1 fungal region was processed using amptk (https://github.com/nextgenusfs/amptk) with dependencies of USEARCH [40] and VSEARCH [41], with commands in Additional file 2. First, R1 and R2 reads were paired, and if pairing was unsuccessful, the R1 reads were retained. After quality filtering with an expected error rate < 1.0, sequences were clustered into OTUs and checked for chimeras de novo with UPARSE [42]. Chimeras were also identified against the ITS database provided within amptk. Taxonomy was assigned through BLAST against a reference database of the mock sequences appended to the UNITE database [43] (release November 20, 2016). OTUs identified as PhiX (*n* = 179) were removed, as were OTUs (*n* = 11) with greater reads in the negative controls than in the samples and an OTU identified as *Amanita phalloides* likely resulting from barcode bleed [44] with other samples sequenced in the same MiSeq library.

The OTU tables were analyzed in R [45] using the vegan [46], Phyloseq [47], ampvis [48], and ggplot2 [49] packages, which created the functionality to graphically summarize the data as heatmaps, constrained ordination, and relative abundance barplots.

Quantitative PCR was done on the BioRad CFX96 Touch Real Time PCR Detection System following protocols previously described [50]. Results are reported as gene copy numbers. Quantitative estimates from the controls (not detected for fungi; ranging from 1500 to 3000 gene copies for bacteria) were subtracted from the sample estimates.

Cultivation was examined from six additional samples (two in the kitchen sink and one in the shower stall) deployed at the second sampling period. To increase the likelihood of acquiring sufficient biomass for genetic isolation, separate samples were used for culturing work than were used for genetic analysis. As with DNA extraction, the sampler was swabbed, and the swab vortexed in 1X phosphate-buffered saline (pH 7.2) to release the cells from the swabs. Aliquots were plated onto 1/10 and full-strength trypticase soy agar (TSA) for bacteria (150 μL onto 10-cm plates) and onto potato dextrose agar (PDA) with ampicillin for fungi (50 μL onto 6-cm plates), and the plates were incubated at 28 °C for 3 days for bacteria and 7 days for fungi. As fungal plates showed no growth, subculturing of 26 individual bacterial colonies (relying on colony morphologies based on phenotypical traits, including surface, texture, color, elevation, and margin) from the six plates was undertaken, and these colonies were subjected to DNA extraction. Taxonomic identities of the bacterial isolates were identified through Sanger sequencing of the full-length 16S region obtained by UC Berkeley’s DNA Sequencing Facility with primers 8F (5′-AGAGTTTGATCCTGGCTCAG-3′) and R1492 (5′-GGTTACCTTGTTAC GACTT-3′) [51, 52]. Reads were assembled using SeqTrace 0.9.0 [53]. Following subculturing for taxonomic identification, the six TSA plates were used to establish mixed bacterial communities for soluble chemical analysis (see below).

Microscopic images were generated to provide a visual characterization of the surfaces. Surfaces were coated with SYTO BC (diluted to 2X) from Molecular Probes (Invitrogen, Carlsbad, CA, USA) and visualized with a Zeiss M1 AxioImager equipped with differential interference contrast (DIC) and a Hamamatsu Orca 03 camera run by BioVision’s iVision software. We also examined the surfaces with environmental scanning electron microscopy (ESEM; Additional file 3).

### Volatile chemical characterization

A proton transfer reaction time-of-flight mass spectrometer (PTR-TOF-MS) was applied to analyze volatile organic chemical (VOC) emissions from coupons and tiles. Two deployed bathroom tiles and four kitchen coupons were studied for each of the two sampling periods. A dynamic chamber approach was used, where VOC-free air from a zero-air generator flushed a 0.5-L glass jar at a flow rate of 0.25 L min^−1^. These jar chambers were equipped with a gas-tight Teflon lid connected using polyetheretherketone (PEEK) fittings and 1.6 mm (1/16″) PEEK tubing to the zero-air generator and to the PTR-TOF-MS. A 2-μm Teflon membrane filter was inserted between the chamber and PTR-TOF-MS instrument to allow only the gas-phase species to enter the instrument. The samples were inserted without touching the internal surface of the jar and placed on a sterile petri dish. A blank control comprising only the glass jar and a sterile petri dish was also sampled. The experimental controls were blank coupons in two replicates and blank tiles in two replicates. The blank coupons and tiles were sterilized with ethanol approximately 1 week before the experiment and left in a sterile petri dish until sampling.

The PTR-TOF-MS instrument sampled each surface individually in a jar chamber for approximately 15 min obtaining full mass scans (1.000 to 500.0 amu) at high time resolution (1 s). The raw time-of-fight (TOF) spectra were pre-processed into count rates and concentrations using the PTRwid software [54]. The instrument was calibrated (to check the transmission efficiency and duty cycle of the TOF detector) using a multicomponent mixture containing a representative mix of volatile organic compounds (VOCs) and microbial VOCs (mVOCs) from Apel-Riemer (Miami, FL, USA) certified to ± 5% accuracy. To account for uncertainty related to a large number of ions representing potentially different structures, an average proton reaction rate constant was used consistently for the entire mass-to-charge (*m/z*) spectrum [55]. The accuracy of such an approach depends on the proton transfer reaction rate coefficient, which is typically accurate to within 30% uncertainty for an individual ion. Because some ions have reaction rate constants that vary in either direction from the default, the uncertainty for the total sum concentration of the ions partially cancels and is approximately 15%. A large number of individual measurements at 1 s (i.e., 900 full *m*/*z* scans per 15 min) ensure high precision and provide for meaningful statistical analysis. During post-processing, the first 5 min after enclosing the sample was rejected and only the subsequent, steady-state concentrations were averaged, including roughly 600 data points per sample. The emission rates were obtained by multiplying the control-subtracted concentrations by the zero-air flow rate. Identical procedures were followed for both sampling periods. As the flow rate was constant and consistent across the sampling points, the concentrations reported here scale with emission rates. An abundance filter of 1 ppt average was applied to automatically reject rare ions that were close to or below the detection limit. Additional criteria excluded internal ions (e.g., related to primary ions from the ion source or water clusters) and known ions which are detected but cannot be accurately quantified by PTR-MS (e.g., COH^+^, NO_2_^+^, NH_3_H^+^). The resulting unified mass list contained 483 and 425 ions for the first and second campaigns, respectively. We note that ion identification was not experimentally confirmed and the reported species are, therefore, putative.

We estimated the contributions that these two surface types would make to total indoor air concentrations of volatile chemicals in a typical house. We therefore modeled the total contribution, given the observed emission rates of particular ion species from these surfaces under our experimental conditions, which would be present in indoor air given certain assumptions and correction factors (described below). The indoor concentration of each ion species was estimated using a single-compartment mass balance model (Eq. 1). The model assumes that the indoor air was well mixed throughout the home and that ventilation was the only means by which the VOCs were removed from the house. The change rate of indoor concentration for ion species *i* (*C*_in_) is a combination of three factors: the emission from indoor source *E* (either kitchen sink or bathroom shower) divided by house volume *V*, plus infiltration of the ion species from outdoor air at its concentration in outdoor air (*C*_out_), minus the removal of its indoor concentration (*C*_in_) by ventilation.

1 $$ \frac{dC_{\mathrm{in},i}}{dt}=\frac{E_i}{V}+{aC}_{\mathrm{out},i}(t)-{aC}_{\mathrm{in},i}(t) $$

The term *a* represents the residence air exchange rate (per hour). By deriving a steady-steady solution (d*C*_in_/d*t* = 0) to Eq. 1, the contribution to indoor concentration *C*_in, ss_ from source E can be estimated using Eq. 2.

2 $$ {C}_{\mathrm{in},\mathrm{ss},i}=\frac{E_i}{aV} $$

In making this estimate, we assumed that the emission rate *E* for each ion species does not change with time, temperature, and relative humidity, and we note that as mVOCs are likely emitted from surfaces throughout a residence, these emission calculations represent a lower-bound estimate. We used Eq. 2 to estimate source-specific contributions to total indoor concentrations for the 15 most abundant VOC species measured from coupon and tiles at the two sampling periods. The sources of interest for this particular analysis were VOC-emitting microorganisms in the kitchen sink and shower stall. Assuming a uniform emission rate from both types of surfaces, an adjustment factor *f* was applied to ion-specific emission rates from coupons and tiles to scale up to an entire kitchen sink and shower stall surface, respectively. The coupon samples had a surface area of 0.0025 m^2^ and tile samplers had 0.0023 m^2^. Commonly used sizes for double bowl kitchen sink (0.84 × 0.56 × 0.23 m, *L* × *W* × *D*) and shower stall (0.81 × 0.81 × 1.83 m, *L* × *W* × *H*) were utilized to compute *f*. Adjustment factors along with typical values of air exchange rate [*a* (h^−1^)] and house volume [*V*] were applied to model the indoor concentration of each ion species from emissions associated with the kitchen sink and the shower stall (Table 1). In these calculations, we assumed that the 160-m^3^ house has one double bowl kitchen sink and one shower stall. Season-specific emission rates and air exchange rates were used for winter (sampling 2) and summer (sampling 1).

**Table 1:** Model parameters for VOC emissions

Parameter	*a* (h^−1^)^a^		V (m^3^)^a^	Kitchen sink *f* (−)^b^	Shower stall *f* (−)^b^
	Summer	Winter			
Value	1.13	0.61	160	550	2200

Parameter values rounded to two or three significant figures

^a^Data for the air exchange rate (*a*) and house volume (V) obtained from Yamamoto et al. [86], using median values of air exchange rate and house volume for Los Angeles County, CA

^b^Correction factor (*f*) used to scale emission rate to an approximate size in a house compared to our sample materials

### Soluble chemicals

Bathroom tiles (two at each sampling period) and kitchen coupons (four at each sampling point) were extracted with methanol for 20 min three times, and the methanol extracts from the same samples were combined and dried down to 500 μL. At each sampling point, two tiles and two coupons that were not exposed to the indoor environment were extracted in the same way as blanks for metabolomics analysis.

To explore the potential of the microorganisms to secrete the chemical compounds observed in the home, we compared our environmental samples with the metabolites of the microorganisms growing in culture. A volume of 1 ml water was washed over each of the six plates of bacterial colonies (two sink communities from kitchen coupons and one bathroom communities from tiles, deployed during S2, grown on full and 1/10 TSA media; see “Microbiota analysis”). Aliquots of 50 μL from each wash were plated onto both the nutrient-rich medium ISP2 agar and on the nutrient-poor medium R2A. The resulting 12 plates were initially incubated at 30 °C overnight and then at room temperature for up to 5 days to mimic indoor growth temperatures. At 1, 3, and 5 days at room temperature, three 5-mm plugs were removed from each culture plate and extracted with 750 μL methanol in an ultrasonic bath for 10 min and left incubating for 1 h at room temperature. Methanolic extracts were centrifuged for 5 min at 14,000 rpm to form pellets from the particles. Supernatants were analyzed via liquid chromatography–mass spectrometry (LC–MS). Non-inoculated R2A and ISP2 agar plugs were extracted in the same way as blanks for metabolomics analysis. Bacterial cultures were stored in 25% glycerol at − 80 °C.

All samples were analyzed using a Thermo Scientific Dionex UltiMate 3000 UHPLC system coupled to Thermo Scientific Q-Exactive Quadrupole-Orbitrap mass spectrometer in heated electrospray ionization (HESI) positive mode. LC separation was performed on a C18 column (50 mm × 2.1 mm, 2.2 μm particle size, Thermo Scientific Acclaim RSLC) using gradient water (0.1% TFA) and methanol (0.1% TFA) as the mobile phase: 10% methanol for 1 min, 10–100% methanol for 10 min, 100% methanol for 2 min, 100–10% for 0.1 min, and 10% methanol for 2.9 min, at a flow rate of 0.4 mL/min. MS analyses were done using two data collection methods: one scanning at a mass-to-charge range of 100–1000 *m*/*z* and another at 1000–2000 *m*/*z*. All samples were analyzed in an electrospray ionization (ESI) positive mode. Full scan parameters were as follows: resolution of 70,000 full width at half maximum (FWHM), automatic gain control (AGC) target of 3 × 10^6^ ions, and a maximum ion injected time (IT) of 100 ms; MS/MS parameters: resolution of 17,500 FWHM, AGC target of 1 × 10^5^ ions, maximum IT of 50 ms, quadrupole isolation window of 4.0 *m*/*z*, and normalized collision energy (NCE) of 35%. Tandem MS was acquired using the data-dependent Top5 method considering precursor ion abundance.

Molecular networking was performed using the GNPS platform (gnps.ucsd.edu) [56], with precursor ion mass tolerance of 2 Da, fragment ion mass tolerance of 0.5 Da, minimum pair cosine of 0.75 and 6 minimum matched fragment ions. GNPS was also used for compound identification, using score threshold cosine of 0.70 and 6 minimum matched peaks. Networks were visualized further using Cytoscape 2.8.0 [57]. Nodes detected in blanks (tiles and coupons not exposed to the indoors environment, ISP2 agar, and R2A) and the methanol extract were removed from the network to facilitate analysis.

## Results

We report observations for the two sampling periods for microbes found on surfaces that we placed in two areas regularly wetted in residences, the kitchen sink and the bathroom shower stall. Our observations include visual and microscopic inspection, analysis of microbes by both DNA and RNA sequencing, analysis of mVOCs emitted from the surfaces, and analysis of soluble chemicals extracted from the sampling surfaces, and from microbes cultivated from the sampling surfaces. The different approaches to study microbial life on household surfaces revealed complementary results and highlighted that much of the chemical ecology of homes remains uncharacterized.

### Microbiota

**Fig. 2:**
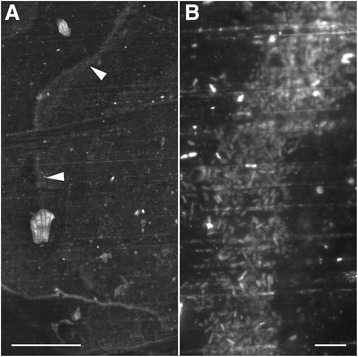
**a** Low-magnification image of bacterial growth on a stainless-steel coupon stained with SYTO BC (green fluorescent nucleic acid stain). Scale bar is 100 μm. Arrows indicate a trail of bacterial growth, viewed in high magnification in panel **b** where the scale bar is 10 μm

To the naked eye, the bathroom tiles appeared to be clean surfaces while the kitchen coupons had visible areas of dried surface film material. Microscopic images showed an analogous summary, where little substance appeared on the tiles, but kitchen coupons showed a rich surface. For example, what appeared to be a trail of dried material when observed at × 10 magnification was seen to be a ribbon of rod-shaped bacteria when viewed at × 100 magnification (Fig. 2).

Table 2 details the quantitative assessment of bacteria and fungi on the different surfaces. Gene copy counts were, on average, × 25 higher for bacteria than fungi on a given surface (*t* test; *p* value < 0.001). The biomass was greater on kitchen coupons than on bathroom tiles (*t* test; *p* = 0.02) and tended to be greater in the first sampling period than the second.

**Table 2:** Quantitative estimates of microbial biomass on kitchen coupons and bathrooms tiles

	Sample	Bacteria	Fungi
Sampling 1			
Kitchen coupons	KC.1	2,300,000 ± 900,000	251,000 ± 94,000
	KC.2	3,800,000 ± 200,000	122,000 ± 5,000
	KC.3	229,000 ± 65,000	17,000 ± 5,000
	KC.4	1,200,000 ± 140,000	226,000 ± 23,000
	Average	1,900,000	154,000
Bathroom tiles	BT.1	36,000 ± 2,000	8,500 ± 4,000
	BT.2	26,700 ± 200	10,200 ± 3,000
	BT.3	200 ± 6	800 ± 500
	Average	21,000	6500
Sampling 2			
Kitchen coupons	KC.5	574,000 ± 46,000	51,300 ± 8,000
	KC.6	887,000 ± 25,000	52,200 ± 6,000
	KC.7	2,870,000 ± 1,500,000	60,900 ± 9,000
	KC.8	404,000 ± 5,000	17,900 ± 6,000
	KC.9	4,620,000 ± 40,000	25,700 ± 2,000
	KC.10	378,000 ± 12,000	19,900 ± 8,000
	Average	1,622,000	38,000
Bathroom tiles	BT.4	780 ± 30	100 ± 80
	BT.5	4,900 ± 200	–^a^
	BT.6	7,800 ± 600	500 ± 100
	BT.7	3,700 ± 100	2200 ± 22,000
	Average	4,300	700

Reported as gene copies/cm^2^ surface, rounded to three significant figures

^a^Below detection limit

**Fig. 3:**
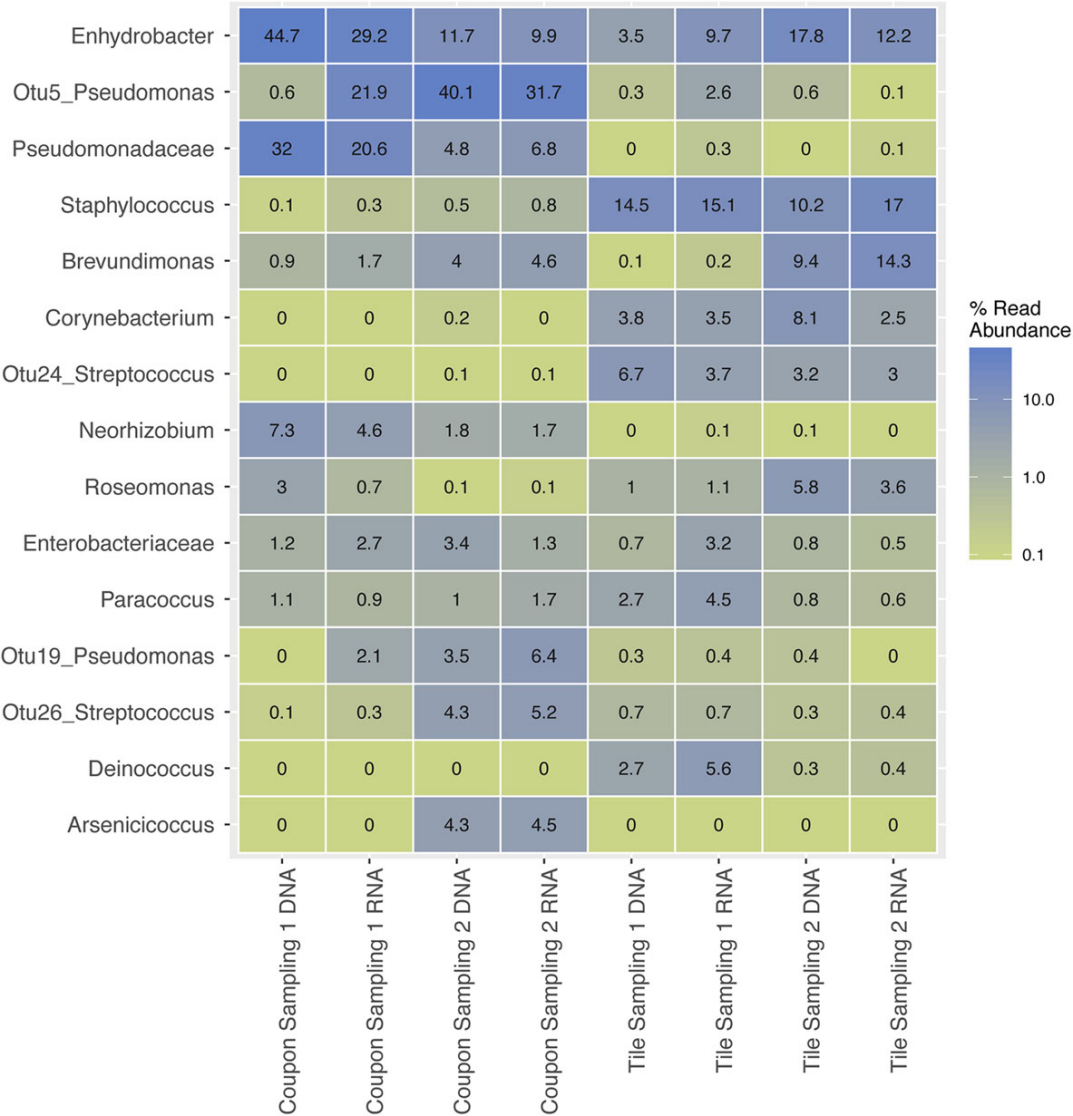
The most abundant bacterial OTUs on kitchen coupons and bathroom tiles in the two sampling campaigns, as detected through amplicon sequencing of DNA and RNA

Microbial community analysis detected approximately 400 each of bacterial and fungal OTUs across the samples. The distribution of the top 15 taxa, representing 60% of the bacterial sequences and over 80% of the fungal sequences, are shown as heatmaps (bacteria—Fig. 3; fungi—Additional file 4). The bacteria *Neorhizobium* and members of the *Pseudomonadaceae* were observed in the kitchen, and other bacteria, *Staphylococcus* and *Corynebacterium*, were much more commonly observed in the shower stall. *Paracoccus* and *Methylobacterium* were found on both materials (the relative abundance of the most abundant bacteria is shown in Additional file 5). Although inter-sample variability in microbial communities was observed (Additional file 5), these differences were less than the variation observed across environments and sampling periods, particularly for the kitchen coupons (Additional file 6). Temporal differences in sampling periods were apparent (Fig. 3 and Additional file 5). For example, *Streptococcus* and *Arsenicicoccus* were seen on the kitchen coupons and *Brevundimonas* on the bathroom tiles only at sampling 2. With regard to fungi, the yeast *Filobasidium magnum* dominated all sample types across both sampling periods. *Knufia epidermidis* was seen in the bathroom tiles but not the kitchen coupons, whereas other fungi, specifically *Candida sake* and *Cladosporium ramotenellum*, were seen in the kitchen. *Olpidium brassicae* was seen on the kitchen coupons from sampling 2. Interestingly, the presence of bacteria and fungi were consistent whether DNA or RNA was targeted for amplification, and RNA reads from fungi were generally low (Fig. 3; Additional file 4). Of the kitchen and bathroom water samples obtained during sampling 1, amplicons were obtained only in the kitchen water sample, and they were dominated by *Methylobacterium* (3 OTUs, 19%) and *Mycobacterium* (1 OTU, 16%).

The 26 bacterial colonies resulting from cells recovered from the six bacterial plates represented 14 species. Three taxa, *Pseudomonas* sp., *P. rhizosphaerae*, and *Staphylococcus epidermidis*, overlapped with the common genera based on direct DNA amplification and sequencing. Four different types of *Methylobacterium* were observed (*M. hispanicum*, *M. populi*, *M. radiotolerans*, and *M. rhodesianum*), and this was a dominant component of the kitchen water sample. The other half of the cultures, most of which are part of the *Bacillales* order, appeared at very low abundance in the amplicons: *Bacillus cereus*, *B. megaterium*, *B. pumilus*, *Gordonia sputi*, *Paenibacillus* sp., *P. odorifer*, and *P. pabuli*.

### Volatile chemicals

**Fig. 4:**
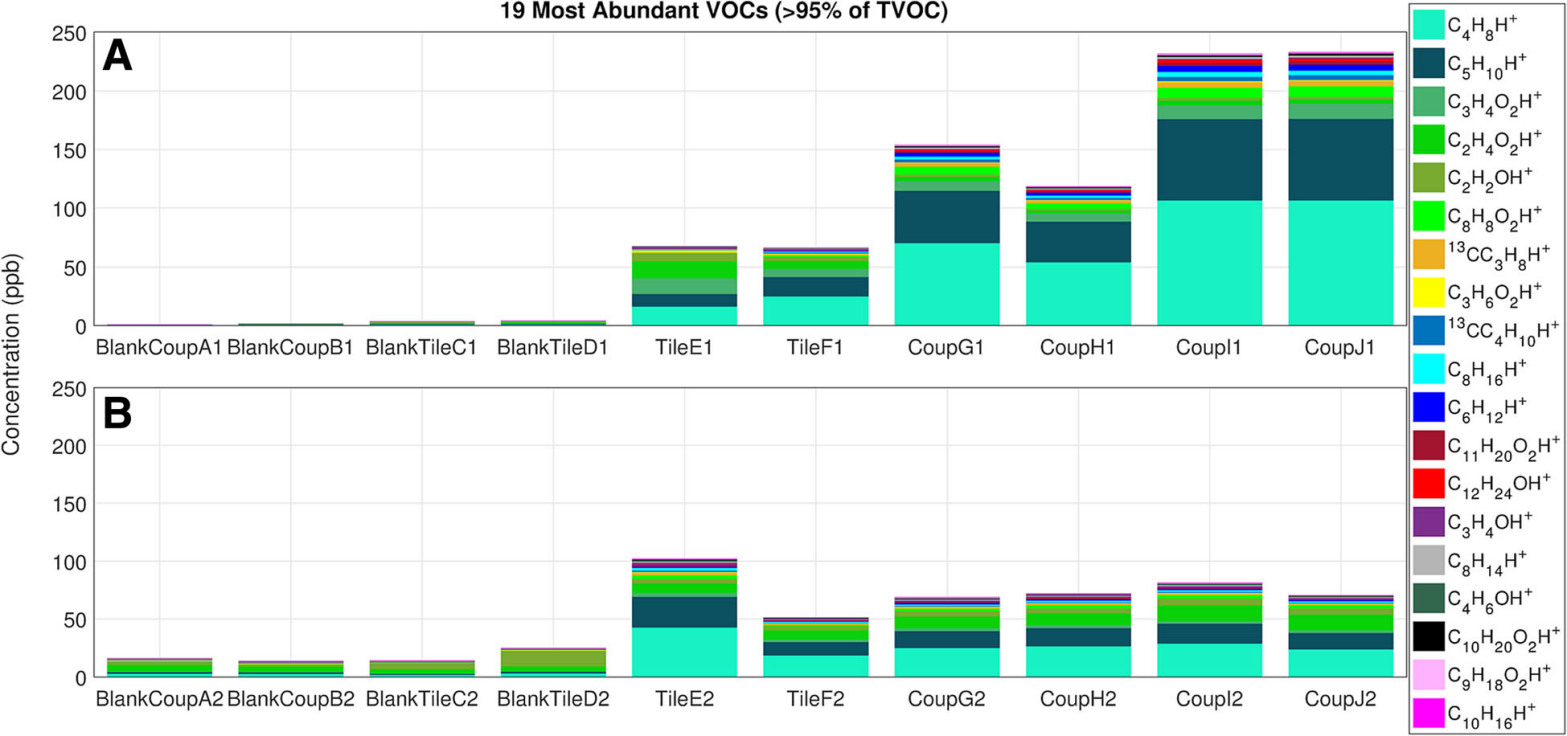
Most abundant VOC ions from blanks, bathroom tiles, and kitchen coupons (“coup”) in sampling 1 (**a**) and sampling 2 (**b**). Letters A–J denote different samples

The 19 most abundant VOCs contributed over 95% of the observed sum of VOCs (∑VOC). The bathroom tiles had similar emissions at both sampling 1 and 2 (Fig. 4); however, total VOC emissions from the kitchen coupons were 3–4× higher during sampling 1 than sampling 2 (*t* test; *p* value = 0.02). The most abundant ions across surfaces and sampling points were C_4_H_8_H^+^ and C_5_H_10_H^+^, which are generic ions representing the sum of alkyl/alkenyl fragments from larger volatile fatty acids (VFAs) and other long-chain VOCs. These ions were well correlated with other alkyl, alkenyl, or alkadienyl fragment ions (i.e., C_6_H_12_H^+^, C_8_H_16_H+, C_10_H_20_H^+^, and C_8_H_14_H^+^) and with the parent and fragment ions of unsaturated short-chain fatty acids (SCFAs) and medium-chain fatty acids (MCFAs).

The three most abundant ions were present at both sampling periods and on both the tiles and coupons. Other ions displayed different patterns between coupons and tiles. For example, emitted molecules had masses consistent with amides, pyridine, dimethylsulfide plus ethanethiol, and benzothiazole, and these molecules were emitted at approximately 10 times higher rates from the kitchen coupons than from the bathroom tiles. Consistent with this observation, there were remarkably fewer compounds specific to the bathroom tiles than the kitchen coupons, and these bathroom compounds corresponded to groups of compounds such as sulfoxides, cyclic amides, and other acids and esters. We define specificity to a particular environment as a presence at orders of magnitude elevation above detection limits. A constrained ordination of the bacterial communities, in which variation in bacteria on samples is constrained by the measured ion data, link two C3 compounds with bathroom tiles (Additional file 6). The compounds consistent with these ions are pyruvaldehyde and acrylic acid/acrylate.

The C_8_H_8_O_2_H^+^ ion, the sixth most abundant ion, is consistent with phenyl acetate and phenyl acetic acid, compounds reported in the literature as being emitted from dermal commensal bacteria such as *Staphylococcus xylosus* [58]. However, the C_8_H_8_O_2_H^+^ formula is structurally nonspecific and it could also have resulted from other compounds such as methyl benzoate or acetophenone, both of which are also known mVOCs [59]. Although in much lower abundance, a sulfur-containing ion (C_4_H_8_OSH^+^) was within the top masses associated with C_8_H_8_O_2_H^+^ on coupons and tiles; it represents contributions from one or more of methional, *S*-methyl thiopropionate, and thioisobutyric acid. The correlation of an abundant ion with a sulfur-containing ion supports the inference that these ions originated from a microbial source.

**Fig. 5:**
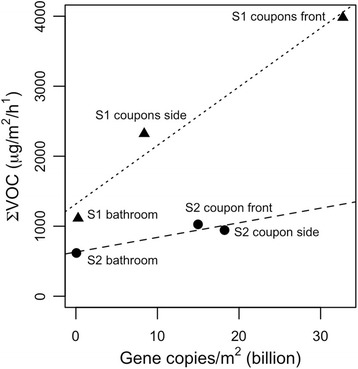
Sum of VOCs, as measured with the PTR-TOF-MS, and microbial gene copies (sum of bacteria and fungi), as estimated with qPCR. “S1” corresponds to sampling period 1 and “S2” to sampling period 2

We sought to explicitly consider the extent to which we could attribute the production of these chemical emissions to microorganisms. To explore whether the quantity of VOCs emitted would track the quantity of microorganisms, we compared the total emission rates of these 19 ions, considered to be an estimate of the sum of all VOCs (∑VOC) with the estimates of microbial biomass based on quantitative PCR. Samples in similar locations were averaged for each of the two sampling periods. For example, VOCs from the front kitchen coupons at sampling 1 were averaged, and these were linked to the average quantitative estimates of microbial biomass from the same set of samples. Each of the two sampling periods was considered separately. The resulting correlations showed that ∑VOC emissions did scale with microbial biomass (Fig. 5), such that, as measured microbial biomass increased, so did the ∑VOC emission rate. Linear regression analysis yielded the following formulae:

$$ \mathrm{Sampling}\ 1:\sum \mathrm{VOC}\ \left(\upmu \mathrm{g}/{\mathrm{m}}^2/\mathrm{h}\right)=8.4\times {10}^{-8}\ \left(\upmu \mathrm{g}/\mathrm{gene}\  \mathrm{copy}/\mathrm{h}\right)\times \mathrm{g}\mathrm{ene}\  \mathrm{copies}/{\mathrm{m}}^2+1300 $$

$$ \mathrm{Sampling}\ 2:\sum \mathrm{VOC}\ \left(\upmu \mathrm{g}/{\mathrm{m}}^2/\mathrm{h}\right)=2.1\times {10}^{-8}\ \left(\upmu \mathrm{g}/\mathrm{gene}\  \mathrm{copy}/\mathrm{h}\right)\times \mathrm{g}\mathrm{ene}\  \mathrm{copies}/{\mathrm{m}}^2+630 $$

The slope represents the production rate of VOCs per gene copy, and the intercept could indicate the background of ∑VOC originating from other sources. Thus, these data indicate a range of ~ 20–80 fg ∑VOC per gene copy per hour, and a background emission in the approximate range of 600–1300 μg ∑VOC per square meter per hour from these samples. We report emissions with two different sets of units, one scaled to gene copies (fg/gene copy/h) and another scaled to surface area (μg/m^2^/h).

**Fig. 6:**
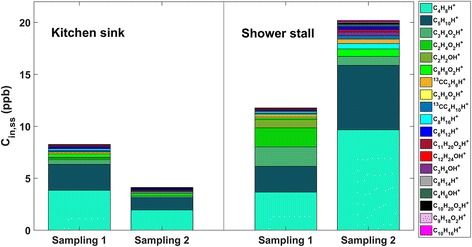
Modeling results of source-specific contributions to indoor concentrations (*C*_in, ss_) from the kitchen sink (left) and shower stall (right)

Results of modeling source-specific contributions to indoor concentrations are shown in Fig. 6, with the raw values included (Additional file 7). The kitchen sink and shower stall are estimated to contribute a combined 20 ppb (55 μg/m^3^) and 24 ppb (70 μg/m^3^) to indoor VOC concentrations for the two sampling periods, respectively. The shower stall showed ~ 1.5× and ~ 4× greater contribution to indoor concentrations than the kitchen sink for the 15 most abundant species, a feature that is attributable to its larger total surface area compared to a kitchen sink. The overall contribution of mVOC emissions to indoor air concentrations showed seasonal differences. For the kitchen surfaces, contributions are predicted to be greater at sampling 1, whereas for the bathroom tiles, contributions are suggested to be greater at sampling 2. This contrasting result is because, in the model, both emission rates and the air exchange rate in the home are expected to play important roles. For the kitchen sink, source emission rates were ~ 4 times higher at sampling 1 than at sampling 2, but the air exchange rate was also ~ 2 times higher. Hence, the predicted concentrations were ~ 2 times higher at the first sampling point. For tiles, the temporal difference in emission rates was small. Therefore, the seasonal difference in air exchange rate would become a dominant factor for tiles, and an increase in VOC abundance associated with these microbial emissions at the conditions of sampling 2 is predicted.

### Soluble chemicals

LC–MS analyses were performed on material samples (tiles and coupons) and on cultures of microbial communities that were isolated from the indoor environment. To identify compounds of microbial origin from the indoor environment, a single molecular network was built using the spectral data obtained from all the indoor samples, spanning both sampling periods and the lab-grown community cultures.

**Fig. 7:**
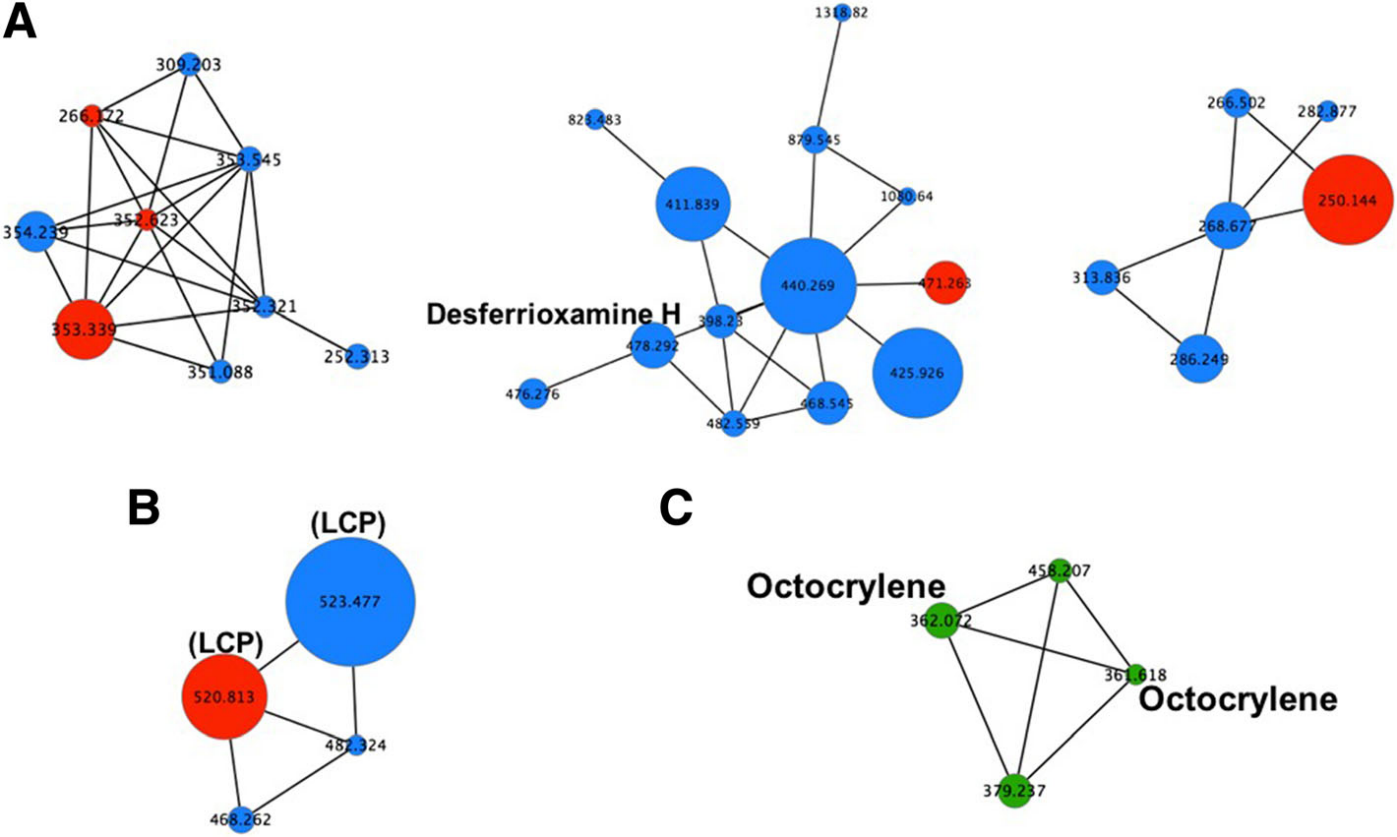
Examples of clusters (**a**–**c**) found in the network constructed using LC-MS data collected from both indoor material samples and microbial cultures. Edges between nodes indicate structural similarity of compounds. Size of the nodes reflects number of spectra found for the same compound and is a measure of compound abundance. Numbers inside each node refer to ion parent mass. Red nodes represent compounds detected in both the culture and indoor material samples. Blue nodes represent compounds found only on microbial cultures. Green nodes represent compounds found only on material samples

Analysis of the network revealed the presence of 2369 detectable compounds. Of these, 2045 of these compounds were only present in microbial cultures (86.3%) and 199 were only present in indoor material samples (8.4%). In all, 81 (3.4%) were detected in both cultures and material samples, representing 28.9% of the total compounds detected in indoor samples. Figure 7 shows examples of clusters present in the overall network that contain compounds detected in both culture and indoor material samples (red nodes). Few of these compounds were specifically identified in terms of their chemical structure. Of the compounds identified, there were examples of molecules potentially synthesized by indoor microbes. For example, lysophosphatidylcholines (LPC) (Fig. 7b), a class of compounds associated with the breakdown of microbial cell walls, were present in both the culture and indoor material samples. LPCs are also commonly found in food, but the fact that this molecule was also seen in the bacterial cultures suggests a potential microbial origin. We also observed the production of a siderophore (iron-chelating compound), desferrioxamine H, and a group of related compounds in the microbial cultures. We note that one compound within this suite (*m*/*z* 471. 26) was found in both the bacterial cultures and from the indoor materials.

Non-microbially produced compounds were also identified. Specifically, octocrylene was found on material samples; this molecule is found in personal hygiene products such as sunscreen (Fig. 7c).

## Discussion

The microbial and chemical signatures reported here provide evidence that surface-borne microorganisms present in the indoor environment play a role in indoor chemistry. There were clear quantitative and qualitative differences in the metabolic profiles between experimental samples and blanks. Microbial metabolism is just one potential source for many VOCs found in indoor environments [60], and it is possible that some of the sampled volatile molecules were derived from absorption and desorption on surface materials. Nevertheless, many of the same compounds seen in experimental samples are also identified when microbes are grown in isolation. For example, in our analysis of soluble compounds, roughly 25% of the compounds observed on the indoor experimental samples were also observed from cultures. Likewise, the SCFAs and MCFAs that were abundant on household surfaces are common VOCs emitted from microbes grown on a variety of substrates [60]. And most notably, VOC concentrations scale with qPCR-measured microbial biomass. In combination, the evidence points firmly in the direction of microbial metabolism being an important source of the VOCs emitted from the sampling surfaces.

One can use these observations to generate hypotheses and inform thinking for subsequent experiments. The results suggest that the detected volatile species are derived predominantly from fatty acid degradation, and these ions include VFAs, alkanes, alkenes, dienes, aldehydes, ketones, and esters. The dominance of these volatile chemicals indicate that fatty acid biodegradation is an important biochemical process occurring on these surfaces, leading to the production of more volatile short- and medium-chain fatty acids. Fatty acids can be the primary metabolites of bacteria for energy and carbon storage [61], and they are the building blocks for membranes and signaling molecules. It has been shown that oxylipin pathways in the bacterium *Pseudomonas aeruginosa* promote biofilm formation when fatty acids are available in the environment [62]. Microbes have the capability to generate short-chain alkanes from fatty acids, a capability that has recently been proposed for industrial production of biofuels [63]. This production was associated with other general bio-oxidation products of fatty acids such as olefins, alcohols, aldehydes, and ketones, which we observed to be emitted from both coupons and tiles. Moreover, the human skin contains cutaneous lipids which can be degraded to short (C3–C5)- and medium (C6–C12)-sized volatile fatty acids [64], and skin oils are known to be hydrolyzed by commensal bacteria [15] as well as oxidized [23, 24].

An interpretation of the linear model that links gene copies on sampled surfaces and with ∑VOCs emitted (Fig. 5) is that the oxidation products from skin oils contribute to a background level of VOCs that microbially mediated breakdown of skin and other long-chain fatty acids increasing further. We hypothesize that fatty acid production and degradation is a general marker of microbial activity rather than one that is specific to given conditions, because these were consistently the common and abundant volatiles, regardless of the specific microbial composition, and increased as microbial biomass increased. That is, microbes showed temporal and spatial variation in response to variable environmental conditions (Fig. 2, S5, S6), but the SCFAs were stable.

We know of no prior studies that have estimated the per-cell or per-gene copy contribution to summed VOC emissions from an indoor surface (Fig. 5). Our estimates of emission rates scaled to surface area are in-line with building wide averages, which have been reported at about 0.5 mg/m^2^/h [65]. Similarly, our estimates of household concentrations based on emissions from these two sources are in the range, albeit toward the lower end, of the previously reported geometric mean concentrations of total VOC, or the sum of selected VOCs. Household concentrations are reported to be in the ~ 100–200 μg/m^3^ range in residences [66–69]. Mean mVOC levels are estimated to be in the approximate range ~ 0.1–12 μg/m^3^, including in problem buildings [60, 70–75]. As has been done in our study, the contribution of VOCs in each study relied on summing a particular set of compounds, and in the case of mVOCs, many previous studies have focused on fungi, whereas our samples were dominated by bacteria. Also, VOCs can originate from many surfaces within a residence, and our models approximate the contribution from two of these surfaces and therefore clearly represent a lower estimate.

Particularly for soluble nonvolatile compounds, it is clear that the identification of environmental chemicals remains an obstacle. In a recent study examining the links between human skin microbiota and metabolites, 3% of the nodes were linked to existing data in the MS/MS spectral libraries [15]. Using a different analysis tool and methods, 4% of the spectra in our samples had hits with known spectra in the GNPS database. There is optimism that the continuous reanalysis of deposited data (so-called living data) will improve classification of molecule detected in existing and future projects [56]. There are also challenges to be met in determining which products originate from microbial processes and in untangling the specific ecological conditions that are associated with these chemical products. In fact, many studies to date have flagged personal care products rather than microbes as exerting prominent influences on the chemical signatures found in indoor environments and on the human skin [15, 76]. It may be that the chemical signal from introduced cleaning and hygiene products overshadows the contributions of metabolites from discrete interactions between microbes and buildings. The role of microbes in modulating this chemical milieu of indoor environments remains unclear.

Cultivation and RNA-based sequencing approaches were used to complement DNA-based surveys for identifying true microbiological residents of these two types of household surfaces. DNA and RNA measurements produced qualitatively, if not quantitatively, similar snapshots of microbial communities, and future efforts will focus on targeting DNA. It is possible that in these regularly wet household surfaces, residual (or “relic” [77]) DNA may not obscure the true biological residents. Nevertheless, it is likely that some taxa were not part of an established community but were rather incidental environmental “tourists.” A striking example would be *Rhizobiaceae*, many of which fix nitrogen in the roots of leguminous plants. Previous culture independent-based studies of kitchen surfaces [28, 29, 31] also observed the presence of *Rhizobiaceae* and a dominance of members of the *Moraxellaceae* family (which sits in the *Pseudomonadales* order and includes the genus *Enhydrobacter*). On the other hand, we did not observe *Micrococcaceae* and *Flavobacteriaceae* to be as common as in previous efforts [29, 31]. These results suggest that while members of the *Moraxellaceae* family may be a common microbial presence in residential kitchens, the remaining community (in our case, 55–90% of the community sequences) may be structured from localized surfaces that can vary over time. Similar to Moen et al. [31], we observed spatial variation in samplers at different locations within the kitchen sink, but this variation was less than other factors, such as house (in their case) and sampling time point (in ours).

Generally, our surveys of the microbiota on household surfaces align with other reports using culture-independent techniques; in that, we observed bacteria that are often plant-associated, including *Neorhizobium* and members of the *Pseudomonadaceae*, predominately in the kitchen and bacteria that are often skin-associated, including *Staphylococcus* and *Corynebacterium*, more commonly in the bathroom [28, 29, 31]. Bacteria likely originating from the premise plumbing system were found on both materials, including *Methylobacterium*, which was observed in the kitchen water sample based on both culturing and sequence-based identification, and *Paracoccus*, which has been observed in premise plumbing and water systems [78, 79]. The *Bacillales*, pseudomonads, staphylococci, and *Methylobacterium* identified through cultivation align with previous culture-based surveys of residences [80–82] but only somewhat with the sequence-based approach. The most abundant identified fungus, *Filobasidium magnum* (synonym, *Cryptococcus magnus*), has been noted in a range of habitats, including an association with humans [83, 84].

Future efforts in building on this study will include setting experimental surfaces in parts of a household that remain dry. We also plan to create artificial communities, drawing on cultured isolates from this study and another residential sampling campaign in which the genomes of the cultured isolates were sequenced [85], to construct microbial communities under experimental control. By manipulating variables such as growth substrate, food source, water availability, and microbial inoculum, we can expand our understanding of the factors that determine the chemical ecology of indoor surfaces.

## Conclusions

An initial examination of the chemical and microbial milieu of household surfaces highlights that there is much to learn about the surfaces of the environments in which we live. Microbial communities can show high temporal and spatial variation in their responses to changing environmental conditions, such as a food source. Taxa found with DNA were also found with RNA, indicating that the microbes were alive on the surfaces and not present as cell fragments containing resilient DNA molecules. Despite the variable microbial results, the indications are that mVOC emissions are more stable in their composition and are predominately associated with the processing of fatty acids. Identification of microbially generated VOCs against a broader background of VOCs in homes remains a challenge, but the VOC output from microbial metabolism should be apparent through appropriate sample controls and integrative measurement techniques. General tracers of microbial life are seen in the soluble compounds. Finer resolution of the chemical interactions among microbes on household surfaces will require a parallel analysis of microbial communities under relevant experimental conditions.

## Additional files

Additional file 1: **Text S1.** Summary of house metadata. Summary of household metadata during sampling 1 and sampling 2, indicating the indoor temperature, occupant-hours of occupancy per day, shower events, and cooking events. (PDF 229 kb)

Additional file 2: **Text S2.** Bioinformatic processing of amplicon sequencing. Commands ran to process to bacterial and fungal sequencing reads, from raw sequences to OTU table and taxonomic identification. (TXT 8 kb)

Additional file 3: **Figure S1.** ESEM images of kitchen coupons and bathroom tiles. Blank and inoculated samples were visualized using environmental scanning electron microscopy (FEI Quanta 3D FEG). The blank stainless steel surface was composed of ridges (A). While interesting structures were observed on the kitchen coupons (B, C), their compositions were unknown. Blank and inoculated ceramic tiles appeared qualitatively similar to each other, and inoculated surfaces are included here. Ceramic tiles contain additives scattered as crystals with different geometries and sizes (D–F) as well as pores, which appear as indentations in the matrix (D, E). There was little observed deposited material on the surface of the bathroom tiles (D–F). Magnification is detailed in each panel. Controls were visualized at high vacuum with 30 kV power, while samples with material were run in ESEM mode and 5 kV power for stainless steel coupons and 10 kV for ceramic tiles. (PDF 5660 kb)

Additional file 4: **Figure S2.** Heatmap of fungi on household surfaces. The most abundant fungal OTUs on kitchen coupons and bathroom tiles in the two sampling campaigns, as detected through amplicon sequencing of DNA and, for kitchen coupons, RNA. (PDF 407 kb)

Additional file 5: **Figure S3.** Barplot of the most abundant OTUs of bacteria on kitchen coupons and bathroom tiles. Relative abundance of the most abundant bacterial OTUs at the genus level (15 OTUs span across 13 genera), based on targeting DNA, across bathroom tiles (BT) and kitchen coupons (KC). Bathroom samples 1–3 (BT.1–BT.3) and kitchen coupons 1–4 (KC.1–KC.4) were collected during sampling 1, while bathroom samples 4–6 (BT.4–BT.6) and kitchen samples 5–10 (KC.5–KC.10) were collecting during sampling 2. (PDF 232 kb)

Additional file 6: **Figure S4.** Constrained ordination of bacterial communities. Variation in bacterial community composition, constrained by the abundance of the most abundant VOC ions observed. Out of the 19 most abundant ions used, 11 of them constrained the ordination in the first two axes, explaining a total of 68.1% of the variation observed. (PDF 227 kb)

Additional file 7: **Table S1.** Modeling results of source-specific contributions of VOCs to indoor concentrations. Raw data for the modeling results are represented in Fig. 6 of the main text. (XLSX 46 kb)

## Abbreviations

amu: Atomic mass unit
ESEM: Environmental scanning electron microscopy
fg: Femtogram (10^−15^ g)
*m/z*: Mass divided by charge number
MCFA: Medium-chain fatty acids
PEEK: Polyetheretherketone
PLFA: Phospholipid-derived fatty acids
ppb: Parts per billion (10^−9^)
SCFA: Short-chain fatty acids
