# Supplementary material for: Microbes and associated soluble and volatile chemicals on periodically wet household surfaces
Source: Microbiome. 2017 Sep 26;5:128. doi: 10.1186/s40168-017-0347-6 (PMC5615633; doi:10.1186/s40168-017-0347-6)
Supplement: Supplementary file 1 — Text S1. Summary of house metadata. Summary of household metadata during sampling 1 and sampling 2, indicating the indoor temperature, occupant-hours of occupancy per day, shower events, and cooking events. (PDF 229 kb) [file 40168_2017_347_MOESM1_ESM.pdf]

Additional file 1: Text S1
Summary of house metadata
Summary of household metadataduring Sampling 1 and Sampling 2.

Summary of metadata results.

	Sampling 1	Sampling 2
Indoor temperature[ǂ] (°C)	23 ± 2	18 ± 1
Occupant-hours per day[#] (h)	23 (21, 24)	22 (20, 23)
Shower events (count)	32	26
Cooking events* (count)	42	23

ǂ: Temperature was measured in the kitchen area (Mean ± standard deviation).
#: Median (1st quartile, 3rd quartile).
*: Cooking events included cooking for lunch or dinner, making applesauce (only during sampling 1), and baking.

Notes:
Cooking event: obtained from occupant maintained logs
Sampling 1: Cooking events include cooking for lunch/dinner (24 times), making applesauce (12 times) and baking (6 times).
Sampling 2: Cooking events include cooking for lunch/dinner (21 times), and baking (2 times).
Shower time: spikes in master bathroom relative humidity (RH)
Shower events were inferred from the change in bathroom relative humidity, which was continuously monitored. We assumed that the short-term spikes in RH were caused by showering. Figures below show master bathroom RH with the spikes marked for both sampling periods. The RH data were obtained from SmartThings RHS sensors mounted in master bathroom.

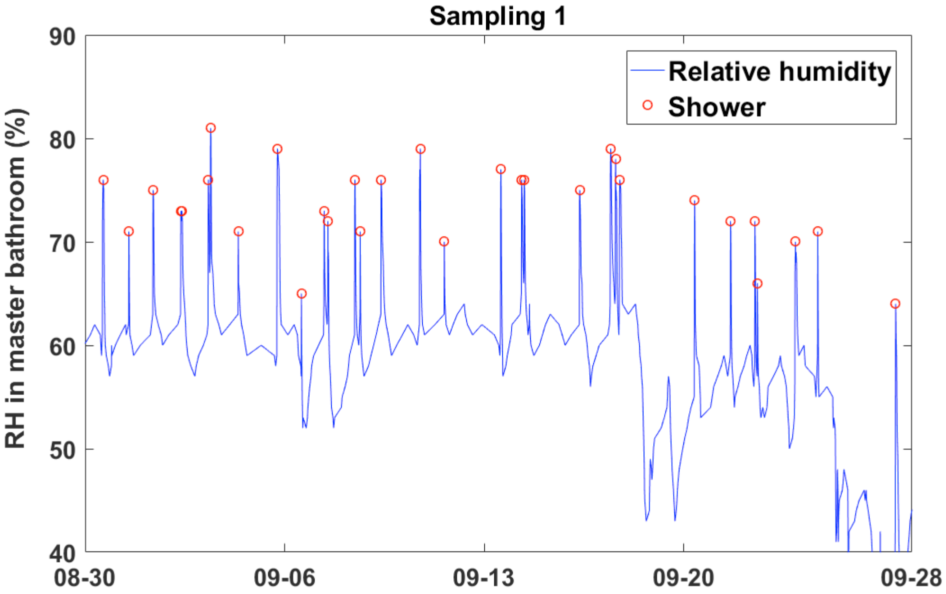
Sampling 1
Relative humidity
Shower
RH in master bathroom (%)
90
80
70
60
50
40
08-30
09-06
09-13
09-20
09-28

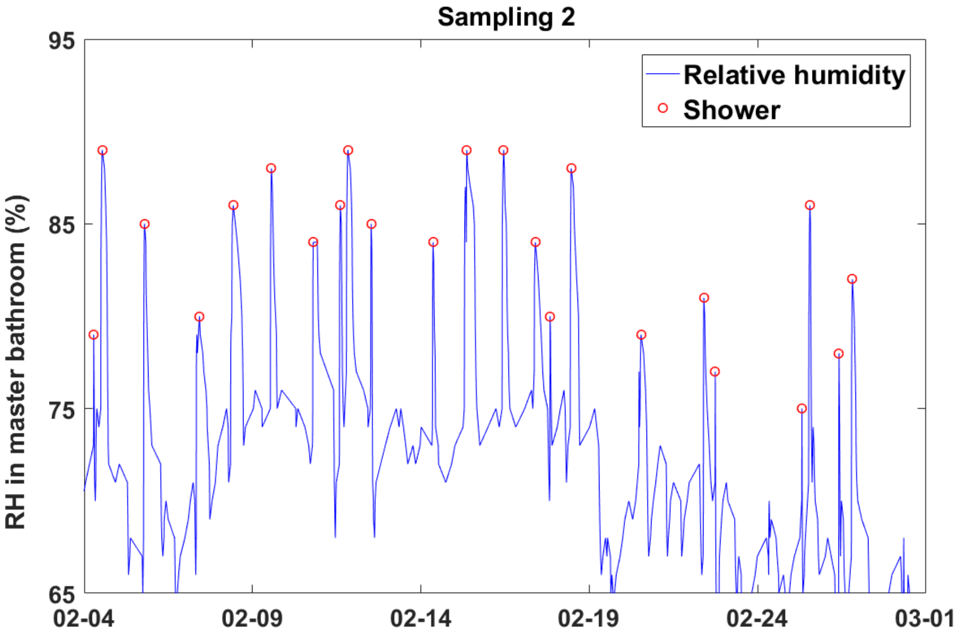
Sampling 2
Relative humidity
Shower
RH in master bathroom (%)
95
85
75
65
02-04
02-09
02-14
02-19
02-24
03-01
