# Supplementary material for: Microbes and associated soluble and volatile chemicals on periodically wet household surfaces
Source: Microbiome. 2017 Sep 26;5:128. doi: 10.1186/s40168-017-0347-6 (PMC5615633; doi:10.1186/s40168-017-0347-6)
Supplement: Supplementary file 3 — Figure S1. ESEM images of kitchen coupons and bathroom tiles. Blank and inoculated samples were visualized using environmental scanning electron microscopy (FEI Quanta 3D FEG). The blank stainless steel surface was composed of ridges (A). While interesting structures were observed on the kitchen coupons (B, C), their compositions were unknown. Blank and inoculated ceramic tiles appeared qualitatively similar to each other, and inoculated surfaces are included here. Ceramic tiles contain additives scattered as crystals with different geometries and sizes (D–F) as well as pores, which appear as indentations in the matrix (D, E). There was little observed deposited material on the surface of the bathroom tiles (D–F). Magnification is detailed in each panel. Controls were visualized at high vacuum with 30 kV power, while samples with material were run in ESEM mode and 5 kV power for stainless steel coupons and 10 kV for ceramic tiles. (PDF 5660 kb) [file 40168_2017_347_MOESM3_ESM.pdf]

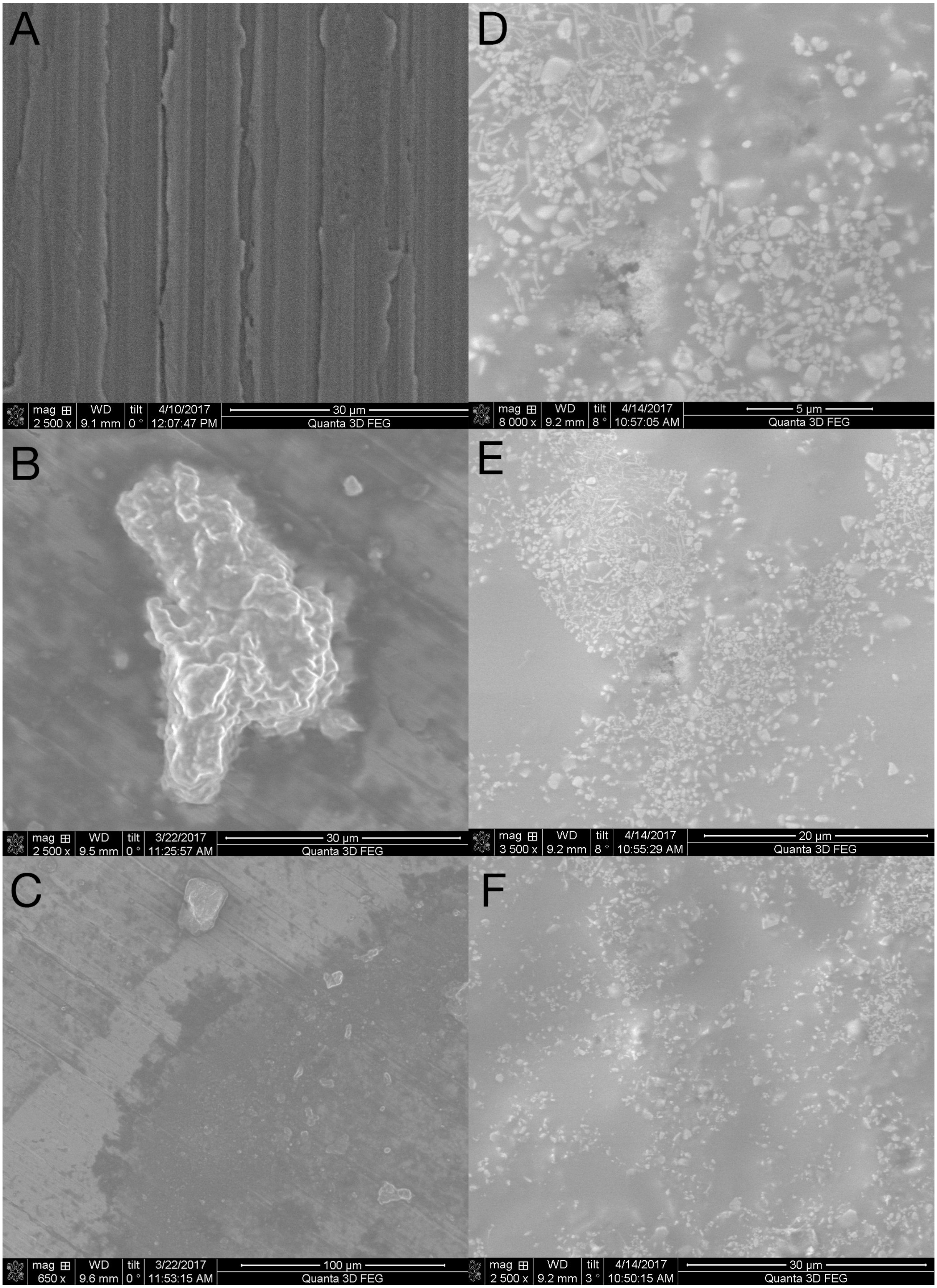
A
mag ⊞ 2 500 x | WD 9.1 mm | tilt 0 ° | 4/10/2017 12:07:47 PM | 30 µm | Quanta 3D FEG
D
mag ⊞ 8 000 x | WD 9.2 mm | tilt 8 ° | 4/14/2017 10:57:05 AM | 5 µm | Quanta 3D FEG
B
mag ⊞ 2 500 x | WD 9.5 mm | tilt 0 ° | 3/22/2017 11:25:57 AM | 30 µm | Quanta 3D FEG
E
mag ⊞ 3 500 x | WD 9.2 mm | tilt 8 ° | 4/14/2017 10:55:29 AM | 20 µm | Quanta 3D FEG
C
mag ⊞ 650 x | WD 9.6 mm | tilt 0 ° | 3/22/2017 11:53:15 AM | 100 µm | Quanta 3D FEG
F
mag ⊞ 2 500 x | WD 9.2 mm | tilt 3 ° | 4/14/2017 10:50:15 AM | 30 µm | Quanta 3D FEG
