# Supplementary figures and images for: Microbes and associated soluble and volatile chemicals on periodically wet household surfaces
Source: Microbiome. 2017 Sep 26;5:128. doi: 10.1186/s40168-017-0347-6 (PMC5615633; doi:10.1186/s40168-017-0347-6)

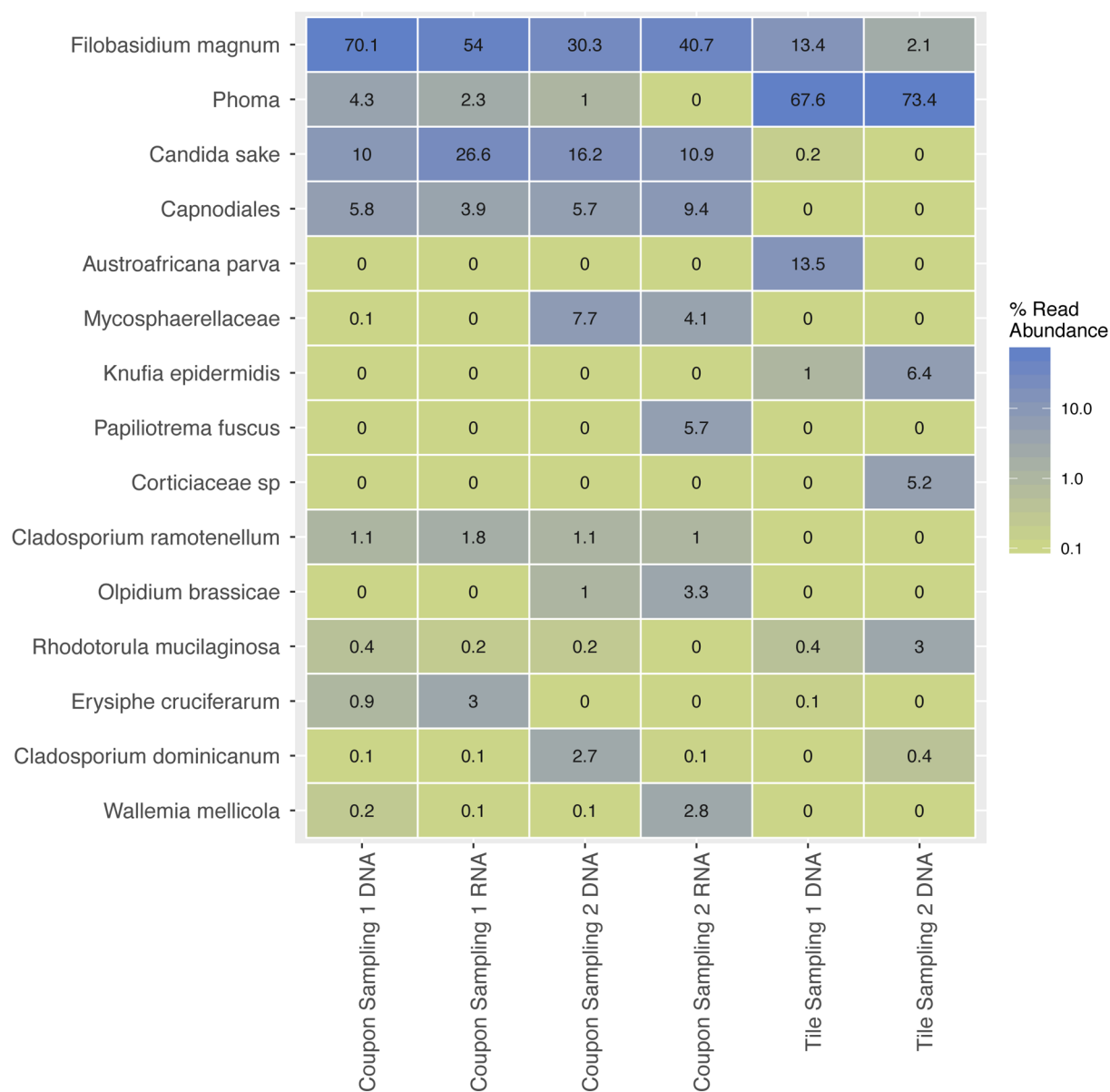

Supplement: Supplementary file 4 — Figure S2. Heatmap of fungi on household surfaces. The most abundant fungal OTUs on kitchen coupons and bathroom tiles in the two sampling campaigns, as detected through amplicon sequencing of DNA and, for kitchen coupons, RNA. (PDF 407 kb) [file 40168_2017_347_MOESM4_ESM.pdf]

Relative Abundance of Top 15 OTUs at the Genus level

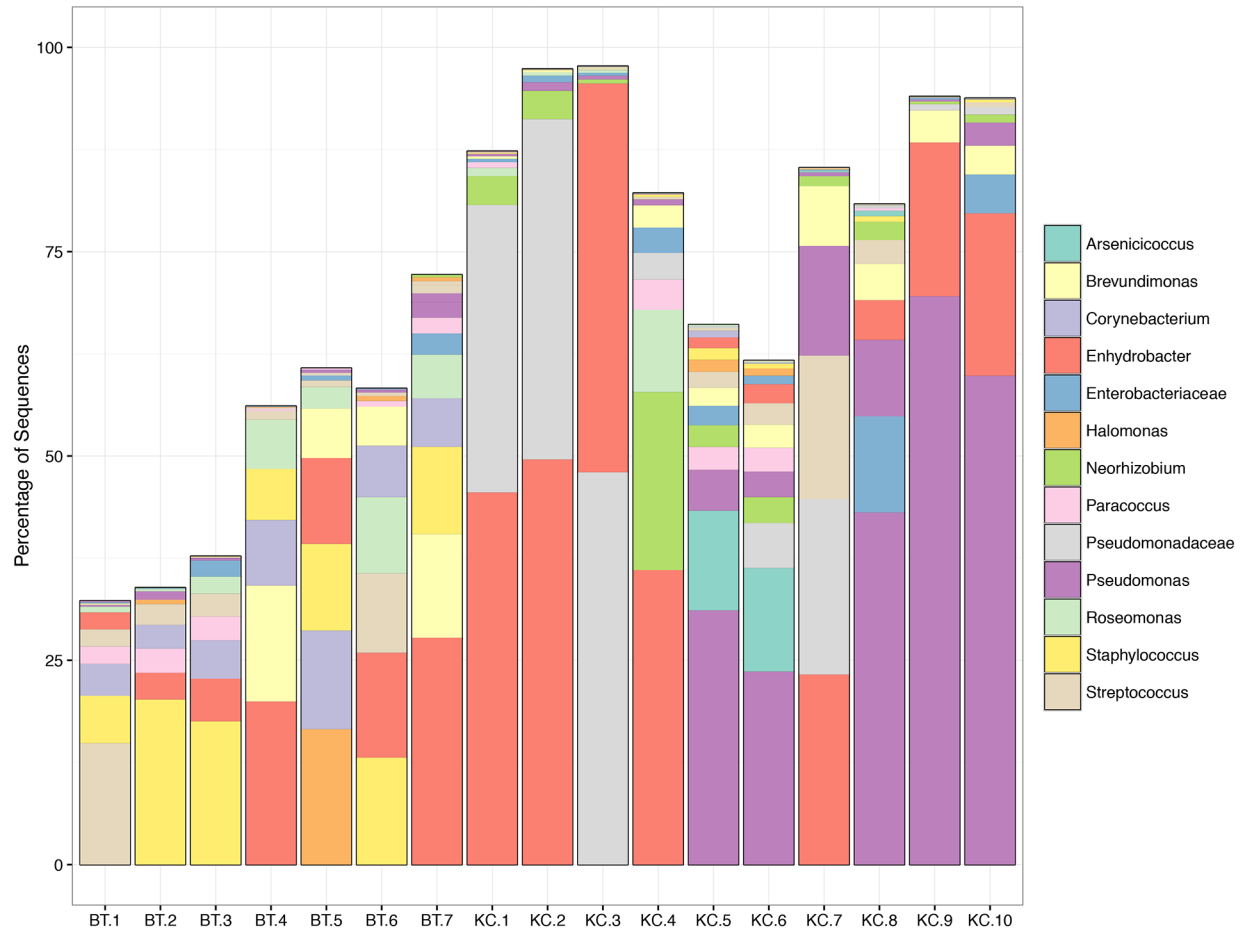

Supplement: Supplementary file 5 — Figure S3. Barplot of the most abundant OTUs of bacteria on kitchen coupons and bathroom tiles. Relative abundance of the most abundant bacterial OTUs at the genus level (15 OTUs span across 13 genera), based on targeting DNA, across bathroom tiles (BT) and kitchen coupons (KC). Bathroom samples 1–3 (BT.1–BT.3) and kitchen coupons 1–4 (KC.1–KC.4) were collected during sampling 1, while bathroom samples 4–6 (BT.4–BT.6) and kitchen samples 5–10 (KC.5–KC.10) were collecting during sampling 2. (PDF 232 kb) [file 40168_2017_347_MOESM5_ESM.pdf]

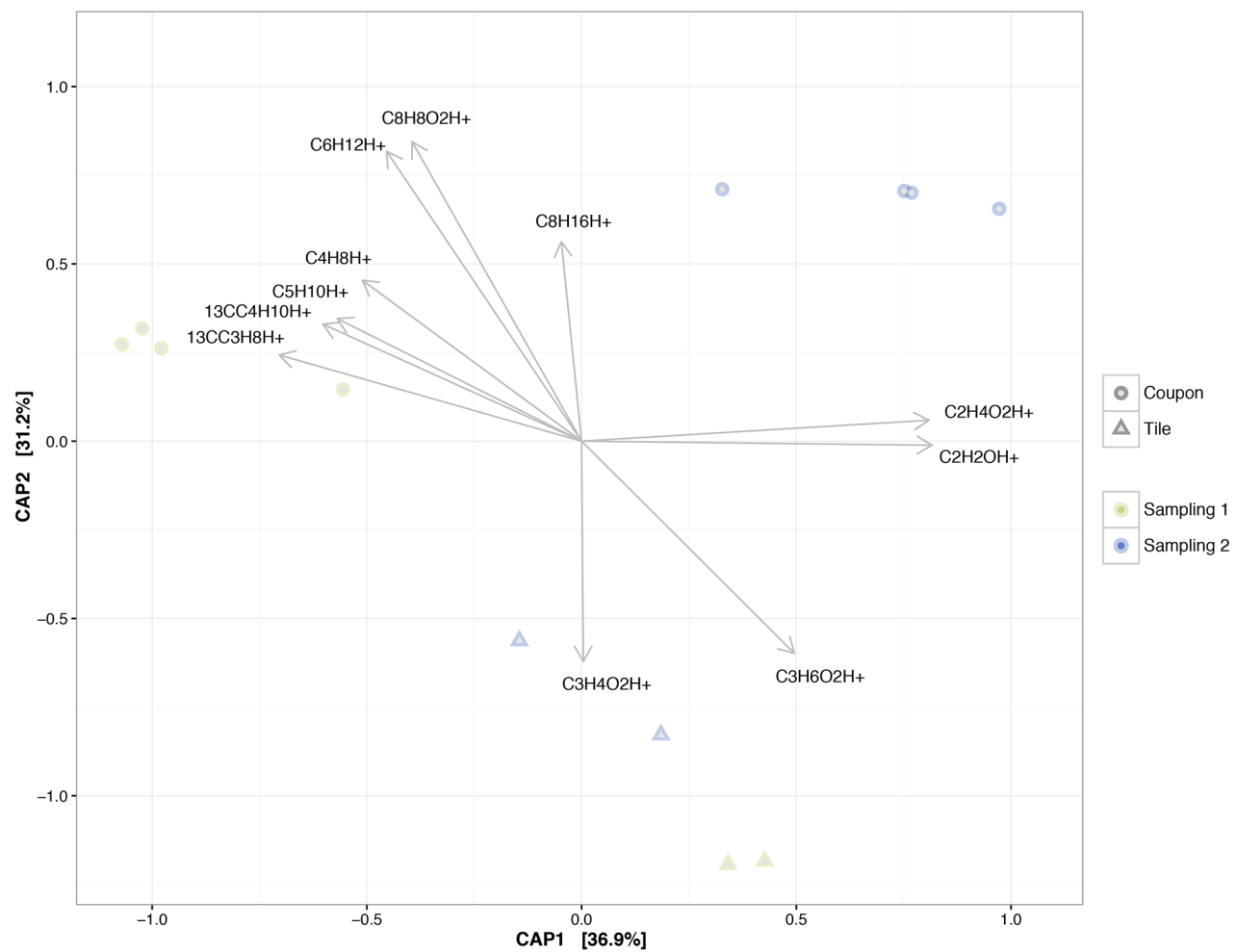

Supplement: Supplementary file 6 — Figure S4. Constrained ordination of bacterial communities. Variation in bacterial community composition, constrained by the abundance of the most abundant VOC ions observed. Out of the 19 most abundant ions used, 11 of them constrained the ordination in the first two axes, explaining a total of 68.1% of the variation observed. (PDF 227 kb) [file 40168_2017_347_MOESM6_ESM.pdf]
